# The effects of laryngeal mask airway versus endotracheal tube on atelectasis in patients undergoing general anesthesia assessed by lung ultrasound: A protocol for a prospective, randomized controlled trial

**DOI:** 10.1371/journal.pone.0273410

**Published:** 2022-09-09

**Authors:** Xuebin Li, Bin Liu, Yaxin Wang, Wei Xiong, Yuan Zhang, Di Bao, Yi Liang, Ling Li, Gaifen Liu, Xu Jin

**Affiliations:** 1 Department of Anesthesiology, Beijing Tiantan Hospital, Capital Medical University, Beijing, China; 2 Department of Neurology, Beijing Tiantan Hospital, Capital Medical University, Beijing, China; 3 China National Clinical Research Center for Neurological Diseases, Beijing, China; University of Texas Medical Branch at Galveston, UNITED STATES

## Abstract

**Background:**

The incidence of atelectasis is high in patients undergoing general anesthesia. This may cause oxygenation impairment and further contribute to postoperative pulmonary complications (PPCs). As important airway management devices for general anesthesia, few studies have compared the effects of laryngeal mask airway (LMA) and endotracheal tube (ETT) on atelectasis. Additionally, lung ultrasound has been increasingly used for bedside atelectasis diagnosis. For the above considerations, this trial is designed to compare the effects of LMA and ETT on atelectasis assessed by lung ultrasound scores, further providing more powerful clinical evidence for perioperative respiratory management of non-laparoscopic elective lower abdominal surgery under general anesthesia.

**Methods:**

This is a prospective, single-center, single-blind, randomized controlled trial. From July 2021 to July 2022, 180 patients undergoing elective non-laparoscopic lower abdominal surgery under general anesthesia will be recruited and randomly divided into the ETT and LMA groups at a ratio of 1:1. The primary outcome is the total atelectasis LUS of 12 lung regions 15 min after the establishment of the artificial airway. The total atelectasis LUS at the end of surgery and 30 min after extubation, oxygenation index, postoperative airway complications, PPCs, and length of stay will be analyzed as secondary indicators.

**Trial registration:**

ClinicalTrials.gov identifier: ChiCTR1900020818. Registered on January 20, 2019. Registered with the name of “Laryngeal mask airway versus endotracheal tube for atelectasis.” URL: https://www.chictr.org.cn/showproj.aspx?proj=35143.

## Background

Atelectasis refers to the decrease in the air volume of one or more pulmonary segments or lobes, and generally occurs in the most dependent part of the lungs, causing many pathophysiological changes such as impaired oxygenation, reduced compliance, and the low ventilation-perfusion ratio [1]. For patients undergoing general anesthesia, atelectasis can occur a few minutes after pre-oxygenation and last until after surgery [2], further contributing to the incidence of other postoperative pulmonary complications (PPCs), increasing mortality, morbidity, and length of stay [3, 4]. The mechanism of atelectasis during general anesthesia has not been fully clarified, and may be related to absorption atelectasis, compression atelectasis, and loss-of-surfactant atelectasis [5, 6]. Airway secretion can be trapped in patients undergoing mechanical ventilation, which may block distal airway obstruction. In this situation, particularly for high FiO_2_, gas inflow is blocked and gas uptake by the blood continues, which can lead to alveolar collapse and promote the formation of atelectasis [7]. The administration of muscle relaxants can lead to cephalic displacement of the diaphragm, and decrease functional residual capacity and lung compliance, to promote the formation of compression atelectasis [8]. In addition, mechanical ventilation may injure alveolar surfactant and contribute to atelectasis [9].

Previous studies have demonstrated that lung-protective ventilation strategies can effectively improve atelectasis and reduce the incidence of PPCs [10–12]. However, few studies have compared the effects of different artificial airways on atelectasis. Endotracheal tube (ETT) has always been considered the gold standard for ensuring a patent airway and adequate ventilation during general anesthesia, but mechanical irritation caused by ETT can lead to reflex bronchoconstriction, and intubation requires more muscle relaxants, which are related to the formation of atelectasis. Laryngeal mask airway (LMA) is increasingly used for ventilation maintenance during general anesthesia as it is more comfortable, causes less irritation and has lower airway resistance [13–17]. Therefore, we hypothesized that using LMA can improve atelectasis in general anesthesia.

Lung ultrasound is a non-invasive bedside tool with real-time imaging ability and without radiation, which is highly suitable for the examination of perioperative atelectasis [18]. In contrast to the clinical diagnosis of atelectasis, the sensitivity of atelectasis diagnosis using lung ultrasound can reach 100%, and mild atelectasis can be detected using lung ultrasound before the clinical manifestations appear [19, 20]. Meanwhile, the accuracy of atelectasis diagnosis using lung ultrasound in both adults and children can exceed 88% [21, 22]. Many studies have used lung ultrasound scores (LUS) to assess atelectasis [10, 23, 24]. We used the sub-region method as described in Sun et al.’s study for quicker and simpler completion of bedside lung ultrasound diagnosis [25].

## Method and analysis

### Trial design

**Fig 1:**
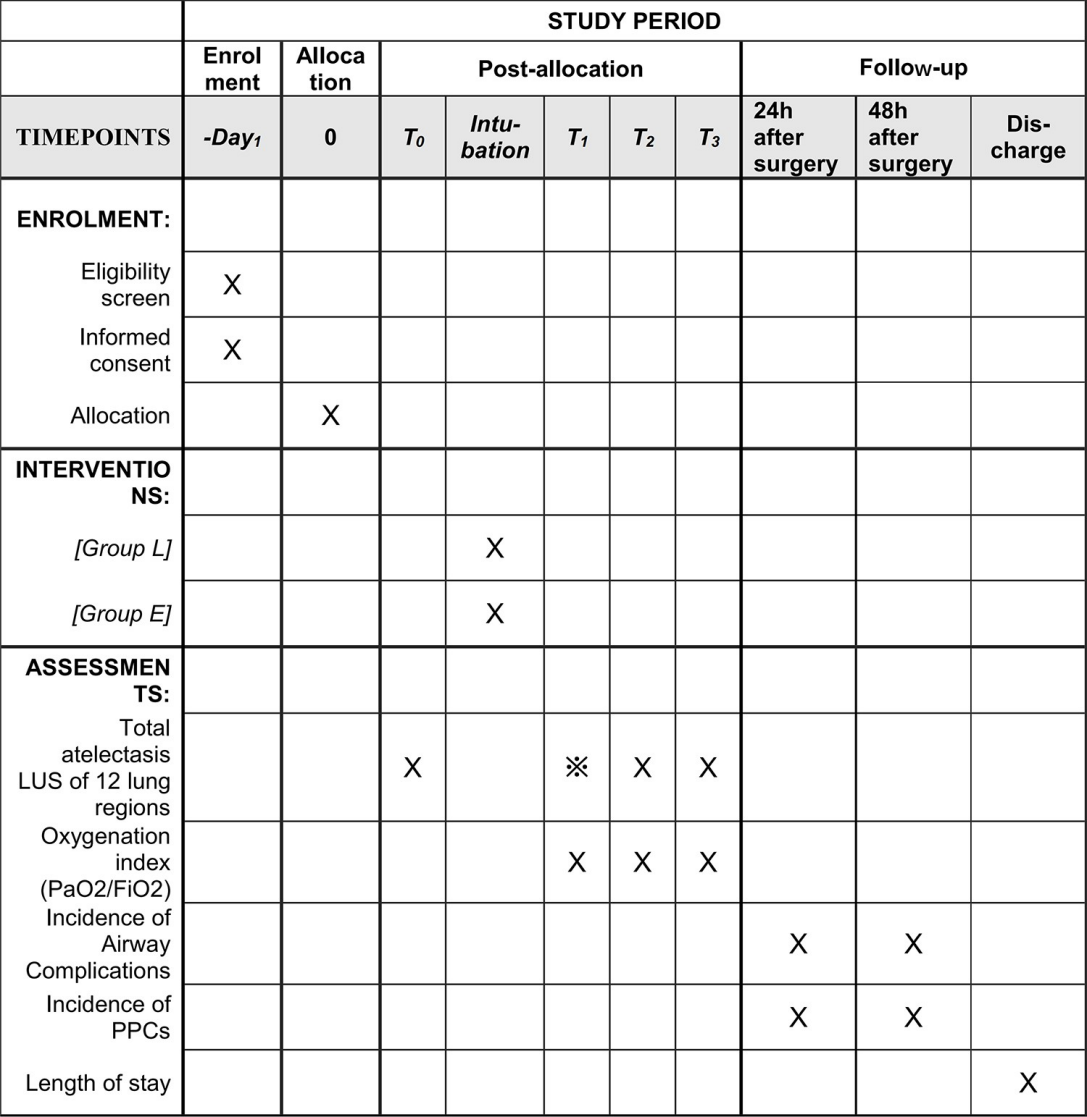
Study schedule. Group L, laryngeal mask airway group; Group E, endotracheal tube group; LUS, lung ultrasound scores; PPCs, postoperative pulmonary complications; T_0_, entering the operating room; T_1_, 15 min after the establishment of the artificial airway; T_2_, the end of the surgery; T_3_, 30 min after extubation.

**Fig 2:**
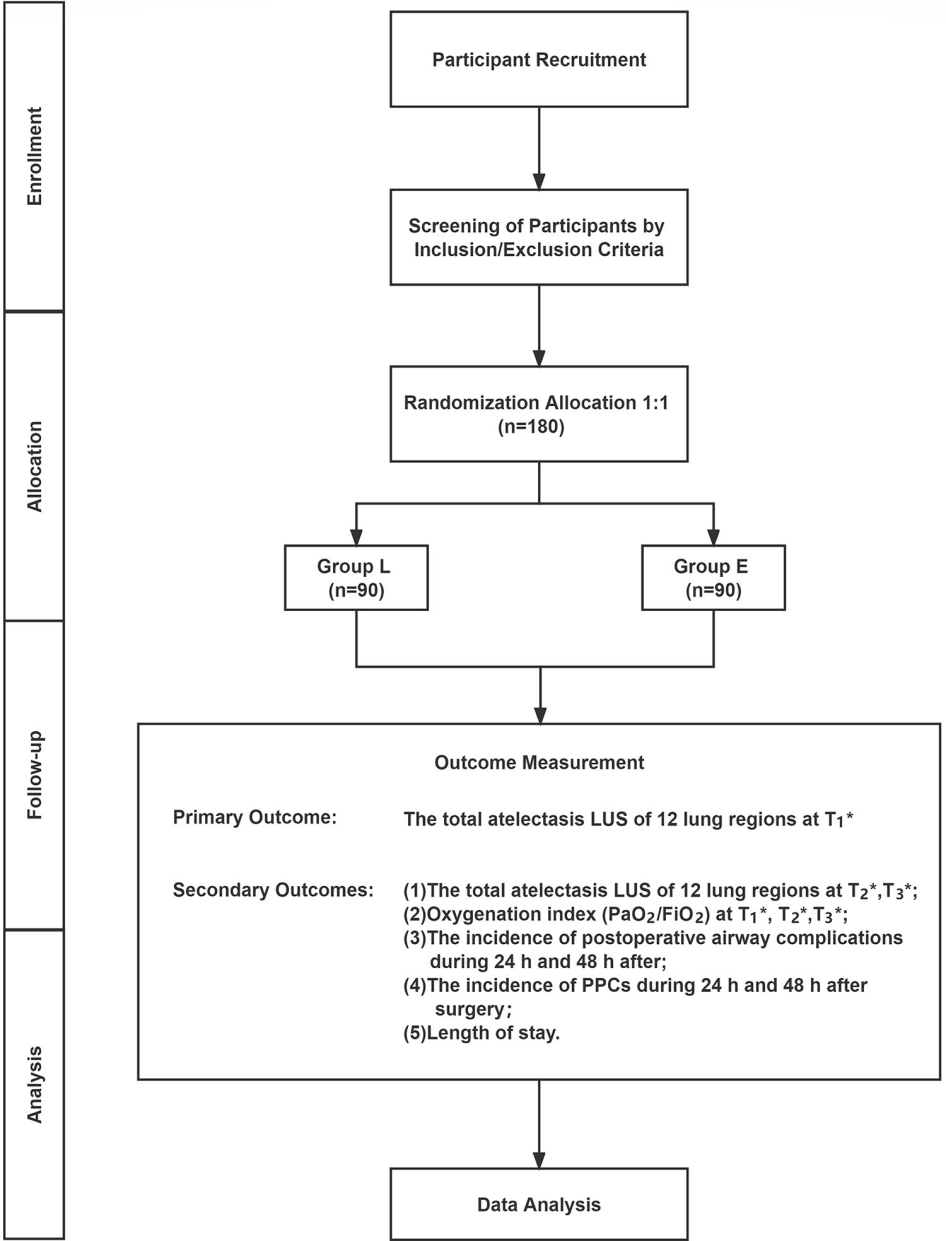
Patients flow diagram. Group L, laryngeal mask airway group; Group E, endotracheal tube group; LUS, lung ultrasound scores; PPCs, postoperative pulmonary complications, *T_1_, 15 min after the establishment of the artificial airway; T_2_, the end of the surgery; T_3_, 30 min after extubation.

This study is a prospective, single-center, single-blinded, randomized controlled trial that aims to explore the effect of LMA and ETT on atelectasis in patients undergoing general anesthesia as assessed using lung ultrasound. The 180 patients who meet the inclusion and exclusion criteria will be randomly allocated to the ETT and LMA groups. This trial is designed according to the updated Consolidated Standards of Reporting Trials statement [26] and conducted at Beijing Tiantan Hospital, Capital Medical University from July 2021 to July 2022. The study schedule is shown in Fig 1, and the patient flow diagram of the study is presented in Fig 2.

### Ethics and dissemination

The trial protocol was approved by the IRB of Beijing Tiantan Hospital, Capital Medical University (approval number: KY 2019-006-01) and strictly adhered to the principles of the latest Declaration of Helsinki. Participants will be asked whether relevant data can be used when signing informed consent. Each patient will be assigned a unique study identifier after they are recruited in the study to conceal and protect private information. This trial does not involve the collection of biological specimens for storage. All paper materials will be stored in the locked cabinet of the anesthesiology department of Beijing Tiantan Hospital. The electronic data will be stored in the database. Only PI has access to all information. If the researchers in charge of the result analysis need access, they need to apply access permission from PI, and access time will be recorded in detail.

Data and information will not be printed or transmitted to auxiliary media. The research results will be disseminated through studies published in peer-reviewed journals and at national and international scientific conferences.

### Eligibility

Patients scheduled for elective non-laparoscopic lower abdominal surgery will be identified as eligible by an independent researcher on the day before the operation. Eligible patients will be informed of the relevant information of the trial and sign the informed consent form by the principal investigator (PI). The inclusion and exclusion criteria are as follows:

#### Inclusion criteria

1. Patients ≥18 years old and ≤ 60 years old;
2. American Society of Anesthesiologists (ASA) physical status of I–III;
3. Patients scheduled for non-laparoscopic elective lower abdominal surgery, including laparotomy with incision below the umbilicus, hysteroscopy, cystoscopy, and ureteroscopy surgery
4. Estimated operation time is < 2 h;
5. Patients and/or their authorized surrogates have signed the informed consent.

#### Exclusion criteria

1. Patients have difficulty with lung ultrasound, such as chest fractures, thoracic deformity, and thoracic surgical history;
2. Patients with a history of respiratory tract infection, smoking, general anesthesia, or mechanical ventilation in the first month before surgery;
3. Patients with cardiac insufficiency (New York Heart Association class IV), chronic renal failure (glomerular filtration rate <30 mL min^−1^·1.73 m^−2^), liver cirrhosis (Child B or C), or pregnancy;
4. Patients with airway abnormalities or reactive airway diseases, including Mallampati classification > II;
5. Patients with BMI>30 kg/m^2^;
6. Patients with the risk of reflux aspiration.

### Intervention

A hundred and eighty eligible patients will be randomly assigned to the LMA group (Group L) and ETT group (Group E) at a 1:1 ratio. The specific scheme is as follows.

Group L: In this group, LMA (Supreme^TM^) are selected for the artificial airway to maintain mechanical ventilation during general anesthesia. LMA size depends on the weight of the patients. After anesthesia induction and 3 min mask ventilation within the 25 cmH_2_O peak pressure, the LMA will be inserted at the appropriate position and fixed properly.

Group E: In this group, ETT are selected for the artificial airway to maintain mechanical ventilation during general anesthesia. Normally, men and women choose a tube with the inner diameter (ID) = 8 mm, and 7.5 mm, respectively. Similar to Group L, ETT will be intubated with appropriate depth and fixed properly in the trachea after anesthesia induction.

The interventions have little potential harm to participants, and there will be no special criteria for modifying the interventions and no relevant compensation. Participants have the right to withdraw from the trial at any time. The coordination center will review the trial conduct situation for every 45 participants. If anesthesiologists in charge think that the LMA cannot ensure the safety of patients, they can replace LMA with ETT at any time during the operation.

### Randomization and blinding

Eligible patients will be randomly allocated into Group L and Group E at a 1:1 ratio, which is determined by the random number sequence generated by Stata 15.1 software (Stata Corp, College Station, TX, USA). The allocation information will be concealed in sealed, opaque envelopes and opened by the anesthesiologist in charge to complete the corresponding intervention. A trained anesthesiologist will complete the lung ultrasound assessment. This anesthesiologist is blinded to the intervention and is not involved in the follow-up and analysis of the results. The group assignment information is also blinded to patients, follow-up researchers, and outcome assessors until all outcomes are statistically analyzed. To ensure clinical safety, the anesthesiologists in charge are unblinded to the allocation information; however, they will not be involved in subsequent procedures such as follow-up and outcome analyses. There were no special criteria for breaking the blinding. If adverse events or serious adverse events (such as reflux aspiration, serious leakage of LMA, severe circulatory instability) occur intraoperatively or postoperatively, PI and the Institutional Review Board (IRB) will be informed immediately. The IRB and PI have the right to break the blinding, if necessary.

### Anesthesia management

Routine monitoring will be performed after participants enter the operating room, including electrocardiogram, non-invasive blood pressure (BP), pulse oxygen saturation (SpO_2_), bispectral index, and temperature. Intravenously administered 0.025–0.075 mg·kg^-1^ midazolam as a premedication. 0.3–0.5 μg·kg^-1^ sufentanil, 1.5–2.5 mg·kg^-1^ propofol, and 0.15 mg·kg^-1^ cisatracurium will be administrated for anesthesia induction. Preoxygenation will last for 3 min with pure oxygen and complete closure of the face-mask will be maintained to ensure P_ET_FiO_2_ ≥ 90%. Then, the anesthesiologist in charge will establish an artificial airway according to the allocation information and cover an asepsic scarf to ensure that the intervention is blinded to the lung ultrasound assessor. Anesthesia maintenance will be administered with a total intravenous infusion of 6–8 mg·kg^-1^·h^-1^ propofol and 0.2–0.3 μg·kg^-1^·min^-1^ remifentanil according to patients’ circulation conditions and anesthesia depth. Mean arterial pressure (MAP) and heart rate (HR) will remain within 30% of the baseline level, and the temperature will remain within 36–37°C during operation. If necessary, vasoactive agents will be used to maintain stable circulation, and the agent type, dosage, and use time will be recorded accurately. A Lung protective ventilation strategy will be applied during the operation and related parameters will be set as follows [27]: tidal volume (Vt) at 6–8 mL·kg^-1^, PEEP = 5 cmH_2_O, with a recruitment maneuver after artificial airway insertion, I:E = 1:1.5, FiO_2_ at 0.4, oxygen flow rate at 1.5 L/min, and respiratory rate (RR) at 12–20 bpm to maintain the end-tidal carbon dioxide partial pressure (P_ET_CO_2_) range from 35 mmHg to 45 mmHg. When the peak airway pressures exceed 25 cmH_2_O, the tidal volume will be turn down to avoid excessive peak pressures. The recruitment maneuver will be performed 3 times, each time with a P_plat_ of 25 cmH_2_O for 15 s. If severe gastric inflation occurs in the LMA group, the recruitment maneuver will be terminated to reduce the risk of aspiration, and the events will be recorded on a case report form (CRF) in detail. All anesthetic infusions will be discontinued at the end of surgery. In addition, patients will receive the same initial postoperative care in the postoperative anesthesia care unit, including incentive spirometry, and administering flurbiprofen axetil or minimal opioids to avoid hypoventilation. The records of incentive spirometry and administrations will be accurately recorded.

### Lung ultrasound

Lung ultrasound assessment will be performed by a blinded trained anesthesiologist. As shown in a previous study, ultrasound scanning will select a 5–12 MHz linear transducer (SonoSite M-Turbo) to parallelly scan all the intercostal space of 12 regions from right to left, anterior to posterior of patients’ chest in the supine position [25]. The six regions are divided by anterior midline, clavicular midline, anterior axillary line, posterior axillary line, and pectoral nipple line in each hemithorax. This anesthesiologist will complete the assessment according to the LUS criteria at the same time as scanning, and the significant images and videos will be recorded.

Atelectasis LUS criteria: 0 indicates normally ventilated areas with lung sliding sign: A-line, or isolated B-lines (< 3); 1 indicates a mild reduction in lung ventilation/moderate loss of lung tissue gasification; B lines (≥ 3) with clear boundary, regular distribution, and spacing ≥ 7 mm, or irregular, clear interval; 2 indicates a severe reduction in lung ventilation/severe lung tissue gasification: multiple coalescent B-lines spaced ≤ 3 mm and continuous fusion; 3 indicates atelectasis/pulmonary consolidation: tissue-like signs, fragment signs, and bronchial inflation signs.

### Outcome measurement

Four time points were significant for outcome measurement, including 15 min after the establishment of the artificial airway (T_1_), the end of surgery (T_2_), and 30 min after extubation (T_3_). In addition, when patients enter the operation room (T_0_), they will accept the lung ultrasound assessment for the basic LUS.

#### Primary outcome

Total atelectasis LUS of 12 lung regions at T_1_.

#### Secondary outcomes

1. Total atelectasis LUS of 12 lung regions at T_2_, T_3_;
2. Oxygenation index (PaO_2_/FiO_2_) at T_1_, T_2_, T_3_;
3. Incidence of postoperative airway complications such as hoarseness, dysphagia, and sore throat during 24 h and 48 h after surgery
4. The incidence of PPCs [28] during 24 h and 48 h after surgery;
5. Length of stay.

### Data collection and follow-up

The indicators that were recorded included baseline data, perioperative data, and follow-up data. All records can be found in the CRF.

Baseline data included: 1) Participants’ general information such as age, height, weight, and vital signs such as basic HR, MAP, RR, and SpO_2_; 2) ASA physical status; 3) Mallampati status; 4) preoperative complications; and 5) arterial blood-gas analysis results.

Perioperative data included: 1) Vital signs (HR, MAP, RR, and SpO_2_) will be recorded every 15 min from the beginning of the operation to 30 min after extubation; 2) Mechanical ventilation parameters (Vt, P_plat_, P_peak_, and dynamic lung compliance [Cdyn] [Cdyn = tidal volume/{PIP-PEEP}]) will be recorded every 15 min from the beginning of the operation to extubation; 3) Atelectasis LUS at T_0_, T_1_, T_2_, and T_3_; 4) Arterial blood-gas analysis results at T_0_, T_1_, T_2_, and T_3_; 4) Administration of perioperative medicines, complete with the type, dosage, way, and time; 5) Several important durations, including the duration of anesthesia, operation, mechanical ventilation duration, and extubation; and 6) Intraoperative quantity of liquid intake, urine, and blood loss.

Follow-up will be performed by the blinded and trained researchers at 24 h and 48 h. Postoperative complications, length of stay, and adverse events during hospitalization will be recorded in detail in the CRF.

### Sample size calculation and statistical analysis

Our pretests estimated that the atelectasis LUS in the ETT group was 8.2±3.87, and we hypothesized that the mean LUS in the LMA group would decrease by 2 scores. The alpha level was set at 0.05, the beta value was set to 0.1, and a dropout rate of 10% was allowed. We calculated that the total sample size required 180 patients (90 patients in each group).

Statistical analysis will be performed using SPSS software (version 23.0; International Business Machines Inc., USA). Continuous variables will be presented as mean ± standard deviation ($\bar{x}$±s), media, or interquartile range, and categorical variables will be presented as numbers (proportion, %). Kolmogorov–Smirnov tests will be performed to detect the normal distribution of continuous variables. After testing for normality of continuous variables, the Student’s t-test, analysis of variance, or the Mann-Whitney U-test will be used for appropriate comparisons between and within groups. Chi-squared and Fisher’s exact tests will be used to analyze categorical variables. All tests will be two-tailed and conducted at a 5% significance level. Statistical significance will be set at P<0.05. The participants missing the primary outcome will be excluded from the outcome analysis, and multiple imputations will be conducted if the unintended drop-out rates are more than 10%. Both intention-to-treat analysis and per protocol analysis will be conducted in this study.

## Discussion

This prospective, single-center, single-blinded, randomized controlled trial aims to compare the effects of LMA and ETT on atelectasis in patients undergoing general anesthesia assessed by lung ultrasound. The study design is based on the following considerations. The influence of general anesthesia on the respiratory system is the focus of clinical attention, and the advantages of lung-protective ventilation strategies in reducing PPCs have been demonstrated. However, few studies have compared the effects of different artificial airways on atelectasis during general anesthesia. Especially for non-laparoscopic abdominal surgery, LMA can ensure the basic ventilation demand with less irritation and less muscle relaxant required for induction, which may be able to improve atelectasis [13–15, 29, 30]. In addition, we selected the total LUS at 15 min after establishing the artificial airway as the primary outcome and comprehensively analyzed the oxygenation index, intraoperative respiratory parameters, PPCs, and postoperative airway complications. Atelectasis appears immediately after induction and can last until after operation [31]. If atelectasis can be improved after induction, it may be beneficial to reduce postoperative atelectasis and PPCs, and further optimize perioperative respiratory management. Moreover, as a new bedside imaging technology, lung ultrasound has been increasingly applied in clinical diagnosis. The high sensitivity of lung ultrasound supports the detection of slight atelectasis before clinical symptoms appear [32]. The subregion method used in this trial is more convenient and rapid in operating room applications and does not disturb normal operating procedures.

This study has some limitations. First, the study population included patients undergoing elective non-laparoscopic lower abdominal surgery, normal BMI, and no serious complications. Therefore, the results cannot be extended to other surgical types or obese individuals. Second, LUS is a relatively subjective score of the blinded assessor, which may cause bias. To solve this problem, the lung ultrasound assessment will be completed by a trained, experienced, blinded anesthesiologist. If the anesthesiologist cannot determine the scores, the most significant images or videos of each scanning site will be recorded and further evaluated by professional ultrasound physicians.

## Supporting information

S1 File SPIRIT-checklist. (DOC)

S2 File Original Chinese research scheme. (PDF)

S3 File Original English research scheme. (PDF)

## Abbreviations

PPCs: postoperative pulmonary complications
ETT: endotracheal tube
LMA: laryngeal mask airway
LUS: lung ultrasound scores
PI: principal investigator
ASA: American Society of Anesthesiologists
IRB: Institutional Review Board
BP: blood pressure
SpO_2_: pulse oxygen saturation
MAP: mean arterial pressure
HR: heart rate
V_t_: tidal volume
RR: respiratory rate
CRF: case report form
P_ET_CO_2_: end-tidal carbon dioxide partial pressure
P_plat_: Plateau pressure
P_peak_: airway peak pressure
Cdyn: dynamic lung compliance
PIP: peak inspiratory pressure

## References

[1] Bendixen HH, Hedley-Whyte J, Laver MB. Impaired Oxygenation in Surgical Patients During General Anesthesia with Controlled Ventilation. A Concept of Atelectasis. The New England journal of medicine. 1963;269:991–996. doi: 10.1056/NEJM196311072691901 14059732

[2] Haaksma ME, Smit JM, Heldeweg MLA, Nooitgedacht JS, de Grooth HJ, Jonkman AH, et al. Extended Lung Ultrasound to Differentiate Between Pneumonia and Atelectasis in Critically Ill Patients: A Diagnostic Accuracy Study. Critical care medicine. 2021. 10.1097/CCM.0000000000005303 34582414

[3] Tusman G, Böhm S, Warner D, Sprung J. Atelectasis and perioperative pulmonary complications in high-risk patients. Current opinion in anaesthesiology. 2012;25(1):1–10. doi: 10.1097/ACO.0b013e32834dd1eb 22113182

[4] Hedenstierna G, Rothen HU. Respiratory function during anesthesia: effects on gas exchange. Comprehensive Physiology. 2012;2(1):69–96. doi: 10.1002/cphy.c080111 23728971

[5] Magnusson L, Spahn D. New concepts of atelectasis during general anaesthesia. British journal of anaesthesia. 2003;91(1):61–72. doi: 10.1093/bja/aeg085 12821566

[6] Hedenstierna G. Oxygen and anesthesia: What lung do we deliver to the post-operative ward? Acta Anaesthesiologica Scandinavica. 2012;56(6):675–685. doi: 10.1111/j.1399-6576.2012.02689.x 22471648

[7] Hedenstierna G, Rothen H. Respiratory function during anesthesia: effects on gas exchange. Comprehensive Physiology. 2012;2(1):69–96. doi: 10.1002/cphy.c080111 23728971

[8] Hedenstierna G, Edmark L. The effects of anesthesia and muscle paralysis on the respiratory system. Intensive care medicine. 2005;31(10):1327–1335. doi: 10.1007/s00134-005-2761-7 16132894

[9] Duggan M, Kavanagh B. Pulmonary atelectasis: a pathogenic perioperative entity. Anesthesiology. 2005;102(4):838–854. doi: 10.1097/00000542-200504000-00021 15791115

[10] Généreux V, Chassé M, Girard F, Massicotte N, Chartrand-Lefebvre C, Girard M. Effects of positive end-expiratory pressure/recruitment manoeuvres compared with zero end-expiratory pressure on atelectasis during open gynaecological surgery as assessed by ultrasonography: a randomised controlled trial. British journal of anaesthesia. 2020;124(1):101–109. doi: 10.1016/j.bja.2019.09.040 31733807

[11] Li X, Jiang D, Jiang Y, Yu H, Zhang M, Jiang J, et al. Comparison of low and high inspiratory oxygen fraction added to lung-protective ventilation on postoperative pulmonary complications after abdominal surgery: A randomized controlled trial. Journal of clinical anesthesia. 2020;67:110009. doi: 10.1016/j.jclinane.2020.110009 32836188

[12] Zhu C, Zhang S, Dong J, Wei R. Effects of positive end-expiratory pressure/recruitment manoeuvres compared with zero end-expiratory pressure on atelectasis in children: A randomised clinical trial. European journal of anaesthesiology. 2021;38(10):1026–1033. doi: 10.1097/EJA.0000000000001451 33534267 PMC8452313

[13] De Rosa S, Messina A, Sorbello M, Rigobello A, Colombo D, Piccolo A, et al. Laryngeal Mask Airway Supreme vs. the Spritztube tracheal cannula in anaesthetised adult patients: A randomised controlled trial. European journal of anaesthesiology. 2019;36(12):955–962. doi: 10.1097/EJA.0000000000001106 31644512

[14] Wang X, Huang K, Yan H, Lan F, Yao D, Li Y, et al. The median effective dose (ED50) of cis-Atracurium for laryngeal mask airway insertion during general Anaesthesia for patients undergoing urinary surgery. BMC anesthesiology. 2020;20(1):68. doi: 10.1186/s12871-020-00982-3 32192431 PMC7081559

[15] Bai F, Li H, Yi M, Yin H, Wu W. The efficacy of different alveolar recruitment maneuvers in holmium laser lithotripsy surgery under general anesthesia using a laryngeal mask. BMC anesthesiology. 2022;22(1):134. doi: 10.1186/s12871-022-01664-y 35501676 PMC9063066

[16] Berry A, Brimacombe J, Keller C, Verghese C. Pulmonary airway resistance with the endotracheal tube versus laryngeal mask airway in paralyzed anesthetized adult patients. Anesthesiology. 1999;90(2):395–397. doi: 10.1097/00000542-199902000-00011 9952143

[17] Ahmad I, Onwochei D, Muldoon S, Keane O, El-Boghdadly K. Airway management research: a systematic review. Anaesthesia. 2019;74(2):225–236. doi: 10.1111/anae.14471 30460982

[18] Monastesse A, Girard F, Massicotte N, Chartrand-Lefebvre C, Girard M. Lung Ultrasonography for the Assessment of Perioperative Atelectasis: A Pilot Feasibility Study. Anesthesia and analgesia. 2017;124(2):494–504. doi: 10.1213/ANE.0000000000001603 27669555

[19] Haaksma M, Smit J, Heldeweg M, Nooitgedacht J, de Grooth H, Jonkman A, et al. Extended Lung Ultrasound to Differentiate Between Pneumonia and Atelectasis in Critically Ill Patients: A Diagnostic Accuracy Study. Critical care medicine. 2022;50(5):750–759. doi: 10.1097/CCM.0000000000005303 34582414

[20] Park M, Jung K, Sim W, Kim D, Chung I, Choi J, et al. Perioperative high inspired oxygen fraction induces atelectasis in patients undergoing abdominal surgery: A randomized controlled trial. Journal of clinical anesthesia. 2021;72:110285. doi: 10.1016/j.jclinane.2021.110285 33838534

[21] Yu X, Zhai Z, Zhao Y, Zhu Z, Tong J, Yan J, et al. Performance of Lung Ultrasound in Detecting Peri-Operative Atelectasis after General Anesthesia. Ultrasound in medicine & biology. 2016;42(12):2775–2784. doi: 10.1016/j.ultrasmedbio.2016.06.010 27639431

[22] Acosta C, Maidana G, Jacovitti D, Belaunzarán A, Cereceda S, Rae E, et al. Accuracy of transthoracic lung ultrasound for diagnosing anesthesia-induced atelectasis in children. Anesthesiology. 2014;120(6):1370–1379. doi: 10.1097/ALN.0000000000000231 24662376

[23] Wu L, Hou Q, Bai J, Zhang J, Sun L, Tan R, et al. Modified Lung Ultrasound Examinations in Assessment and Monitoring of Positive End-Expiratory Pressure-Induced Lung Reaeration in Young Children With Congenital Heart Disease Under General Anesthesia. Pediatric critical care medicine: a journal of the Society of Critical Care Medicine and the World Federation of Pediatric Intensive and Critical Care Societies. 2019;20(5):442–449. doi: 10.1097/PCC.0000000000001865 31058784

[24] Song I, Kim E, Lee J, Ro S, Kim H, Kim J. Effects of an alveolar recruitment manoeuvre guided by lung ultrasound on anaesthesia-induced atelectasis in infants: a randomised, controlled trial. Anaesthesia. 2017;72(2):214–222. doi: 10.1111/anae.13713 27804117

[25] Sun L, Wu L, Zhang K, Tan R, Bai J, Zhang M, et al. Lung ultrasound evaluation of incremental PEEP recruitment maneuver in children undergoing cardiac surgery. Pediatric pulmonology. 2020;55(5):1273–1281. doi: 10.1002/ppul.24720 32150673

[26] Schulz K, Altman D, Moher D. CONSORT 2010 Statement: updated guidelines for reporting parallel group randomised trials. BMC medicine. 2010;8:18. doi: 10.1186/1741-7015-8-18 20334633 PMC2860339

[27] Young C, Harris E, Vacchiano C, Bodnar S, Bukowy B, Elliott R, et al. Lung-protective ventilation for the surgical patient: international expert panel-based consensus recommendations. British journal of anaesthesia. 2019;123(6):898–913. doi: 10.1016/j.bja.2019.08.017 31587835

[28] Abbott T, Fowler A, Pelosi P, Gama de Abreu M, Møller A, Canet J, et al. A systematic review and consensus definitions for standardised end-points in perioperative medicine: pulmonary complications. British journal of anaesthesia. 2018;120(5):1066–1079. doi: 10.1016/j.bja.2018.02.007 29661384

[29] Lee Y, Wang J, Yang Y, Chen A, Lai H. Midazolam vs ondansetron for preventing postoperative nausea and vomiting: a randomised controlled trial. Anaesthesia. 2007;62(1):18–22. doi: 10.1111/j.1365-2044.2006.04895.x 17156222

[30] Verma S, Sharma S. Effectiveness of Proseal laryngeal mask airway and laryngeal tube suction in elective non-laparoscopic surgeries of up to ninety minutes duration: A prospective, randomized study. Journal of anaesthesiology, clinical pharmacology. 2018;34(1):58–61. doi: 10.4103/joacp.JOACP_101_16 29643624 PMC5885450

[31] Gunnarsson L, Tokics L, Gustavsson H, Hedenstierna G. Influence of age on atelectasis formation and gas exchange impairment during general anaesthesia. British journal of anaesthesia. 1991;66(4):423–432. doi: 10.1093/bja/66.4.423 2025468

[32] Hansell L, Milross M, Delaney A, Tian DH, Ntoumenopoulos G. Lung ultrasound has greater accuracy than conventional respiratory assessment tools for the diagnosis of pleural effusion, lung consolidation and collapse: a systematic review. Journal of physiotherapy. 2021;67(1):41–48. doi: 10.1016/j.jphys.2020.12.002 33353830

