# Supplementary material for: The effects of laryngeal mask airway versus endotracheal tube on atelectasis in patients undergoing general anesthesia assessed by lung ultrasound: A protocol for a prospective, randomized controlled trial
Source: PLoS One. 2022 Sep 9;17(9):e0273410. doi: 10.1371/journal.pone.0273410 (PMC9462747; doi:10.1371/journal.pone.0273410)
Supplement: S1 File — (DOC) [file pone.0273410.s001.doc]

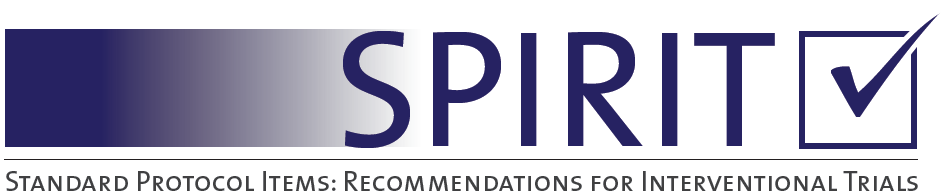


SPIRIT 2013 Checklist: Recommended items to address in a clinical trial protocol and related documents*

| Section/item | ItemNo | Description |
| --- | --- | --- |
| **Administrative information** | | |
| Title | 1 | Descriptive title identifying the study design, population, interventions, and, if applicable, trial acronym  On line 1 page 1, The Effects of Laryngeal Mask Airway Versus Endotracheal Tube on Atelectasis in Patients Undergoing General Anesthesia Assessed by Lung Ultrasound: A protocol for a prospective, randomized controlled trial |
| Trial registration | 2a | Trial identifier and registry name. If not yet registered, name of intended registry  On line 39 page 3, ClinicalTrials.gov identifier: ChiCTR1900020818. Registered on January 20, 2019. Registered with the name of “Laryngeal mask airway versus endotracheal tube for atelectasis.” |
|  | 2b | All items from the World Health Organization Trial Registration Data Set  N/A |
| Protocol version | 3 | Date and version identifier  On line 327 page 18, The protocol version was 3.0, registered on January 10, 2019. The enrolment of patients in this trial started on July 15, 2021, and the trial is still ongoing. |
| Funding | 4 | Sources and types of financial, material, and other support  On line 337 page 19, This trial was supported by a grant from the Beijing Dongcheng District Outstanding Talent Funding Project (No.2019DCT-M-17). |
| Roles and responsibilities | 5a | Names, affiliations, and roles of protocol contributors  On line 5 page 1, Xuebin Li^1#^, Bin Liu^1#^, Yaxin Wang^1#^, Wei Xiong^1^, Yuan Zhang^1^, Di Bao^1^, Yi Liang^1^, Ling Li^1^, Gaifen Liu^2.3^, Xu Jin^1*^  On line 10 page 1,   1. Department of Anesthesiology, Beijing Tiantan Hospital, Capital Medical University, Beijing 100070, China. 2. Department of Neurology, Beijing Tiantan Hospital, Capital Medical University, Beijing 100070, China. 3. China National Clinical Research Center for Neurological Diseases, Beijing 100070, China.   On line 343 page 19, Xu Jin and Xuebin Li conceived and designed the study. Bin Liu and Yaxin Wang were responsible for writing the manuscript. Yi Liang was responsible for registration and ethical applications. Wei Xiong contributed to patient recruitment. Yuan Zhang was responsible for clinical anesthesia. Di Bao was responsible for data collection and follow-up. Gaifen Liu and Ling Li were responsible for data management and statistical analyses. All named authors have read the manuscript and have agreed to its publication. |
|  | 5b | Name and contact information for the trial sponsor  N/A, the trial is funded by government projects. |
|  | 5c | Role of study sponsor and funders, if any, in study design; collection, management, analysis, and interpretation of data; writing of the report; and the decision to submit the report for publication, including whether they will have ultimate authority over any of these activities  On line 337 page 19, This trial was supported by a grant from the Beijing Dongcheng District Outstanding Talent Funding Project (No.2019DCT-M-17), which did not play any part in the study design, management, analysis, or interpretation of the data. |
|  | 5d | Composition, roles, and responsibilities of the coordinating centre, steering committee, endpoint adjudication committee, data management team, and other individuals or groups overseeing the trial, if applicable (see Item 21a for data monitoring committee)  On line 143 page 10, The coordination center will review the trial conduct situation for every 45 participants.  On line 331 page 19, The data management committee consists of PI, and two statisticians will provide monitoring, including verifying the progress of the study and all observational data. Quality control was performed at all stages of the data processing. |
| Introduction |  |  |
| Background and rationale | 6a | Description of research question and justification for undertaking the trial, including summary of relevant studies (published and unpublished) examining benefits and harms for each intervention  On line 22 page 1, The incidence of atelectasis is high in patients undergoing general anesthesia. This may further damage oxygenation and cause postoperative pulmonary complications (PPCs). The advantages of lung-protective ventilation strategy in reducing atelectasis have been confirmed; however, with the increasing application of laryngeal mask airway (LMA), there is hardly any relevant study to compare the effects of LMA and endotracheal tube (ETT) on atelectasis to date. Additionally, lung ultrasound has been increasingly used for bedside atelectasis diagnosis. For the above considerations, we designed this trial to compare the effects of LMA and ETT on atelectasis assessed using lung ultrasound scores (LUS). This study provides more powerful clinical evidence for perioperative respiratory management. |
|  | 6b | Explanation for choice of comparators  On line 62 page 3, The endotracheal tube (ETT) has always been considered the gold standard for ensuring a patent airway and adequate ventilation during general anesthesia. |
| Objectives | 7 | Specific objectives or hypotheses  On line 260 page 15, This prospective, single-center, single-blinded, randomized controlled trial aimed to compare the effects of LMA and ETT on atelectasis in patients undergoing general anesthesia assessed by lung ultrasound and to compare the prognosis of LMA and ETT. |
| Trial design | 8 | Description of trial design including type of trial (eg, parallel group, crossover, factorial, single group), allocation ratio, and framework (eg, superiority, equivalence, noninferiority, exploratory)  On line 84 page 4, This study is a prospective, single-center, single-blinded, randomized controlled trial that aims to explore the effect of LMA and ETT on atelectasis in patients undergoing general anesthesia as assessed using lung ultrasound. |
| Methods: Participants, interventions, and outcomes | | |
| Study setting | 9 | Description of study settings (eg, community clinic, academic hospital) and list of countries where data will be collected. Reference to where list of study sites can be obtained  On line 88 page 5, This trial is designed according to the updated Consolidated Standards of Reporting Trials statement[24] and conducted at Beijing Tiantan Hospital, Capital Medical University from July 2021 to July 2022. |
| Eligibility criteria | 10 | Inclusion and exclusion criteria for participants. If applicable, eligibility criteria for study centres and individuals who will perform the interventions (eg, surgeons, psychotherapists)  On line 110 page 8, “Inclusion criteria” and “Exclusion criteria” part. |
| Interventions | 11a | Interventions for each group with sufficient detail to allow replication, including how and when they will be administered  On line 128 page 9, Intervention part. |
|  | 11b | Criteria for discontinuing or modifying allocated interventions for a given trial participant (eg, drug dose change in response to harms, participant request, or improving/worsening disease)  On line 141 page 9, The interventions have little potential harm to participants, and there will be no special criteria for modifying the interventions and no relevant compensation. |
|  | 11c | Strategies to improve adherence to intervention protocols, and any procedures for monitoring adherence (eg, drug tablet return, laboratory tests)  On line 143 page 10, The coordination center will review the trial conduct situation for every 45 participants. |
|  | 11d | Relevant concomitant care and interventions that are permitted or prohibited during the trial  On line 164 page 11, “Anesthesia management” section |
| Outcomes | 12 | Primary, secondary, and other outcomes, including the specific measurement variable (eg, systolic blood pressure), analysis metric (eg, change from baseline, final value, time to event), method of aggregation (eg, median, proportion), and time point for each outcome. Explanation of the clinical relevance of chosen efficacy and harm outcomes is strongly recommended  On line 208 page 13, “Outcome measurement” section. |
| Participant timeline | 13 | Time schedule of enrolment, interventions (including any run-ins and washouts), assessments, and visits for participants. A schematic diagram is highly recommended (see Figure)  On page 6, Figure 1 Patients flow diagram of the study  On page 7, Table 1, Study schedule. |
| Sample size | 14 | Estimated number of participants needed to achieve study objectives and how it was determined, including clinical and statistical assumptions supporting any sample size calculations  On line 242 page 14, Our pre-tests estimated that the atelectasis LUS in ETT was 8.2±3.87. The difference in LUS, at least for a score of 2, is considered clinically significant. The alpha level was set at 0.05, the beta value was set to 0.1, and allowing for a dropout rate of 10%. We calculated that the total sample size required 180 patients (90 patients in each group) |
| Recruitment | 15 | Strategies for achieving adequate participant enrolment to reach target sample size  On line 123 page 6, This trial is designed according to the updated Consolidated Standards of Reporting Trials statement and conducted at Beijing Tiantan Hospital, Capital Medical University from July 2021 to July 2022. |
| **Methods: Assignment of interventions (for controlled trials)** | | |
| Allocation: |  |  |
| Sequence generation | 16a | Method of generating the allocation sequence (eg, computer-generated random numbers), and list of any factors for stratification. To reduce predictability of a random sequence, details of any planned restriction (eg, blocking) should be provided in a separate document that is unavailable to those who enrol participants or assign interventions  On line 148 page 10, Eligible patients will be randomly allocated into Group L and Group E at a 1:1 ratio, which is determined by the random number sequence generated by Stata 15.1 software (Stata Corp, College Station, TX, USA). |
| Allocation concealment mechanism | 16b | Mechanism of implementing the allocation sequence (eg, central telephone; sequentially numbered, opaque, sealed envelopes), describing any steps to conceal the sequence until interventions are assigned  On line 150 page 10, The allocation information will be concealed in sealed, opaque envelopes and opened by the anesthesiologist in charge to complete the corresponding intervention. |
| Implementation | 16c | Who will generate the allocation sequence, who will enrol participants, and who will assign participants to interventions  On line 135 page 8, Patients scheduled for elective lower abdominal surgery will be identified as eligible by an independent researcher on the day before the operation.  On line 148 page 10, Eligible patients will be randomly allocated into Group L and Group E at a 1:1 ratio, which is determined by the random number sequence generated by Stata 15.1 software (Stata Corp, College Station, TX, USA). The allocation information will be concealed in sealed, opaque envelopes and opened by the anesthesiologist in charge to complete the corresponding intervention. |
| Blinding (masking) | 17a | Who will be blinded after assignment to interventions (eg, trial participants, care providers, outcome assessors, data analysts), and how  On line 224 page 11, A trained anesthesiologist will complete the lung ultrasound assessment. This anesthesiologist is blinded to the intervention and is not involved in the follow-up and analysis of the results. The group assignment information is also blinded to patients, follow-up researchers, and outcome assessors until all outcomes are statistically analyzed. To ensure clinical safety, the anesthesiologists in charge are unblinded to the allocation information; however, they will not be involved in subsequent procedures such as follow-up and outcome analyses. |
|  | 17b | If blinded, circumstances under which unblinding is permissible, and procedure for revealing a participant’s allocated intervention during the trial  On line 159 page 10, There were no special criteria for breaking the blinding. If adverse events or serious adverse events (such as reflux aspiration, serious leakage of LMA, severe circulatory instability) occur intraoperatively or postoperatively, PI and the Institutional Review Board (IRB) will be informed immediately. The IRB and PI have the right to break the blinding, if necessary. |
| **Methods: Data collection, management, and analysis** | | |
| Data collection methods | 18a | Plans for assessment and collection of outcome, baseline, and other trial data, including any related processes to promote data quality (eg, duplicate measurements, training of assessors) and a description of study instruments (eg, questionnaires, laboratory tests) along with their reliability and validity, if known. Reference to where data collection forms can be found, if not in the protocol  On line 221 page 13, “Data collection and Follow-up” section |
|  | 18b | Plans to promote participant retention and complete follow-up, including list of any outcome data to be collected for participants who discontinue or deviate from intervention protocols  On line 238 page 14, Follow-up will be performed by the blinded and trained researchers at 24 h and 48 h. Postoperative complications, length of stay, and adverse events during hospitalization will be recorded in detail in the CRF. |
| Data management | 19 | Plans for data entry, coding, security, and storage, including any related processes to promote data quality (eg, double data entry; range checks for data values). Reference to where details of data management procedures can be found, if not in the protocol  On line 293 page 17, Each patient will be assigned a unique study identifier after they are recruited in the study to conceal and protect private information. This trial does not involve the collection of biological specimens for storage. All paper materials will be stored in the locked cabinet of the anesthesiology department of Beijing Tiantan Hospital. The electronic data will be stored in the database. Only PI has access to all information. If the researchers in charge of the result analysis need access, they need to apply access permission from PI, and access time will be recorded in detail. |
| Statistical methods | 20a | Statistical methods for analysing primary and secondary outcomes. Reference to where other details of the statistical analysis plan can be found, if not in the protocol  On line 247 page 15, Statistical analysis will be performed using SPSS software (version 23.0; International Business Machines Inc., USA). Continuous variables will be presented as mean ± standard deviation (x±s) media, or interquartile range, and categorical variables will be presented as numbers (proportion, %). Kolmogorov–Smirnov tests will be performed to detect the normal distribution of continuous variables. After testing for normality of continuous variables, the Student’s t-test, analysis of variance, or the Mann-Whitney U-test will be used for appropriate comparisons between and within groups. Chi-squared and Fisher’s exact tests will be used to analyze categorical variables. All tests will be two-tailed and conducted at a 5% significance level. Statistical significance will be set at P<0.05. The participants missing the primary outcome will be excluded from the outcome analysis, and multiple imputations will be conducted if the unintended drop-out rates are more than 10%. |
|  | 20b | Methods for any additional analyses (eg, subgroup and adjusted analyses)  N/A, no additional analyse in this study. |
|  | 20c | Definition of analysis population relating to protocol non-adherence (eg, as randomised analysis), and any statistical methods to handle missing data (eg, multiple imputation)  On line 256 page 15, The participants missing the primary outcome will be excluded from the outcome analysis, and multiple imputations will be conducted if the unintended drop-out rates are more than 10%. |
| **Methods: Monitoring** | | |
| Data monitoring | 21a | Composition of data monitoring committee (DMC); summary of its role and reporting structure; statement of whether it is independent from the sponsor and competing interests; and reference to where further details about its charter can be found, if not in the protocol. Alternatively, an explanation of why a DMC is not needed  On line 331 page 19, The data management committee consists of PI, and two statisticians will provide monitoring, including verifying the progress of the study and all observational data. Quality control was performed at all stages of the data processing. There is no interim analysis plan for our study because of the low-risk intervention and short-term research periods. |
|  | 21b | Description of any interim analyses and stopping guidelines, including who will have access to these interim results and make the final decision to terminate the trial  On line 333 page 19, There is no interim analysis plan for our study because of the low-risk intervention and short-term research periods. |
| Harms | 22 | Plans for collecting, assessing, reporting, and managing solicited and spontaneously reported adverse events and other unintended effects of trial interventions or trial conduct  On line 159 page 10, If adverse events or serious adverse events (such as reflux aspiration, serious leakage of LMA, severe circulatory instability) occur intraoperatively or postoperatively, PI and the Institutional Review Board (IRB) will be informed immediately. The IRB and PI have the right to break the blinding, if necessary. |
| Auditing | 23 | Frequency and procedures for auditing trial conduct, if any, and whether the process will be independent from investigators and the sponsor  On line 143 page 10, The coordination center will review the trial conduct situation for every 45 participants.  On line 331 page 19, The data management committee consists of PI, and two statisticians will provide monitoring, including verifying the progress of the study and all observational data. Quality control was performed at all stages of the data processing. |
| Ethics and dissemination | | |
| Research ethics approval | 24 | Plans for seeking research ethics committee/institutional review board (REC/IRB) approval  On line 290 page 17, The trial protocol was approved by the IRB of Beijing Tiantan Hospital, Capital Medical University (approval number: KY 2019-006-01) and strictly adhered to the principles of the latest Declaration of Helsinki. |
| Protocol amendments | 25 | Plans for communicating important protocol modifications (eg, changes to eligibility criteria, outcomes, analyses) to relevant parties (eg, investigators, REC/IRBs, trial participants, trial registries, journals, regulators)  On line 327 page 18, Any deviations from the protocol will be recorded and reported to the IRB. The PI will revise the protocol and update the new version of the clinical trial registration website. |
| Consent or assent | 26a | Who will obtain informed consent or assent from potential trial participants or authorised surrogates, and how (see Item 32)  On line 106 page 8, Eligible patients will be informed of the relevant information of the trial and sign the informed consent form by the principal investigator (PI). |
|  | 26b | Additional consent provisions for collection and use of participant data and biological specimens in ancillary studies, if applicable  On line 292 page 17, Participants will be asked whether relevant data can be used when signing informed consent. This trial does not involve the collection of biological specimens for storage. |
| Confidentiality | 27 | How personal information about potential and enrolled participants will be collected, shared, and maintained in order to protect confidentiality before, during, and after the trial  On line 293 page 17, Each patient will be assigned a unique study identifier after they are recruited in the study to conceal and protect private information. This trial does not involve the collection of biological specimens for storage. All paper materials will be stored in the locked cabinet of the anesthesiology department of Beijing Tiantan Hospital. The electronic data will be stored in the database. Only PI has access to all information. If the researchers in charge of the result analysis need access, they need to apply access permission from PI, and access time will be recorded in detail. |
| Declaration of interests | 28 | Financial and other competing interests for principal investigators for the overall trial and each study site    On line 340 page 19, The authors declare that they have no competing interests. |
| Access to data | 29 | Statement of who will have access to the final trial dataset, and disclosure of contractual agreements that limit such access for investigators    On line 298 page 17, Only PI has access to all information. If the researchers in charge of the result analysis need access, they need to apply access permission from PI, and access time will be recorded in detail. |
| Ancillary and post-trial care | 30 | Provisions, if any, for ancillary and post-trial care, and for compensation to those who suffer harm from trial participation  On line 141 page 9, The interventions have little potential harm to participants, and there will be no special criteria for modifying the interventions and no relevant compensation. |
| Dissemination policy | 31a | Plans for investigators and sponsor to communicate trial results to participants, healthcare professionals, the public, and other relevant groups (eg, via publication, reporting in results databases, or other data sharing arrangements), including any publication restrictions  On line 301 page 17, Data and information will not be printed or transmitted to auxiliary media. The research results will be disseminated through studies published in peer-reviewed journals and at national and international scientific conferences. |
|  | 31b | Authorship eligibility guidelines and any intended use of professional writers  On line 341 page 19, “Authors’ contributions” section |
|  | 31c | Plans, if any, for granting public access to the full protocol, participant-level dataset, and statistical code  N/A |
| Appendices |  |  |
| Informed consent materials | 32 | Model consent form and other related documentation given to participants and authorised surrogates  The informed consent documents can be provided if they are required. |
| Biological specimens | 33 | Plans for collection, laboratory evaluation, and storage of biological specimens for genetic or molecular analysis in the current trial and for future use in ancillary studies, if applicable  On line 295 page 17, This trial does not involve the collection of biological specimens for storage. |

*It is strongly recommended that this checklist be read in conjunction with the SPIRIT 2013 Explanation & Elaboration for important clarification on the items. Amendments to the protocol should be tracked and dated. The SPIRIT checklist is copyrighted by the SPIRIT Group under the Creative Commons “[Attribution-NonCommercial-NoDerivs 3.0 Unported](http://www.creativecommons.org/licenses/by-nc-nd/3.0/)” license.
