# Supplementary material for: The effects of laryngeal mask airway versus endotracheal tube on atelectasis in patients undergoing general anesthesia assessed by lung ultrasound: A protocol for a prospective, randomized controlled trial
Source: PLoS One. 2022 Sep 9;17(9):e0273410. doi: 10.1371/journal.pone.0273410 (PMC9462747; doi:10.1371/journal.pone.0273410)
Supplement: S2 File — (PDF) [file pone.0273410.s002.pdf]

项目来源与编号：

喉罩对比气管插管用于全麻手术肺不张的随机对照研究

研究方案

The Effects of Laryngeal Mask Airway Versus Endotracheal Tube on Atelectasis in Patients Undergoing General Anesthesia: A protocol for a prospective, randomized controlled trial

所属项目名称：喉罩对比气管插管用于全麻手术肺不张的随机对照研究

项目负责人/承担科室：金旭/麻醉科

课题委托单位：首都医科大学附属北京天坛医院麻醉科

课题承担单位：首都医科大学附属北京天坛医院麻醉科

课题主持单位：首都医科大学附属北京天坛医院麻醉科

研究年限：2019 年 2 月－2022 年 12 月

版本号：V3.0

版本日期：2019 年 1 月 1 日

PI / Department: Xu Jin / Department of Anesthesiology

Main research institutes: Beijing Tiantan Hospital, Capital Medical University

Programming term: 2019.2-2022.12

Version number: V 3.0

Version date: 2019.1.1

方 案 摘 要

**项目名称**	喉罩对比气管插管用于全麻手术肺不张的随机对照研究
**研究目的**	目前，大量的文献研究表明，术后肺部并发症严重影响手术病人的术后转归，其中肺不张占第二位。肺不张主要发生在全身麻醉诱导建立气道后。在此类手术时，建立不同的气道可能对肺不张的发生有影响，确定哪一种人工气道发生肺不张的比例小，成为医务人员面对此类手术必须要考虑的问题。<br>本课题在保证患者安全的前提下在并争得患者知情同意的前提下，选择择期进行泌尿，下腹部，下肢手术患者手术的成年患者，进行临床相关数据的采集和观察处理。根据临床及远期随访数据，总结出最佳的预防及治疗术后方案，以供推广。
**研究设计**	前瞻随机对照双盲研究
**病例总数**	180例
**病例选择**	入选标准<br>1）年龄 18-60 岁；2）ASA 1-3 级；3）择期泌尿，下腹部，下肢手术患者手术的成年患者；4）预计手术时间＜2h；5）患者及其法定家属已签署知情同意书。
	排除标准<br>1）胸部骨折、手术史等应用肺部超声困难者；2）术前一月上呼吸道感染史，吸烟史，术前一月全麻或机械通气史；3）心功能不全或合并心肺疾病；4）Mallampati 分级＞II级；5）BMI＞30 kg/m^2；6）有反流误吸可能。
**治疗方案**	患者再采用随机数字法进行随机分组，分为喉罩组和生理盐水组。<br>喉罩组（L 组）：患者插管前吸 100%氧气。诱导后预充氧时采用机械通气，潮气量 6-8 ml/kg，呼吸频率 12-20 次/分钟，吸入氧浓度 100%。插入喉罩后，立刻机械通气，在机械通气下判断喉罩对位情况。患者入室后，采集肺部 B 超数据。气管插管后 15min 采集肺部 B 超数据。术毕拔除喉罩前采集 B 超数据 。<br>气管插管组（E 组）患者插管前吸 100%氧气。诱导后预充氧时采用机械通气，潮气量 8 ml/kg，呼吸频率 12-16 次/分钟，吸入氧浓度 100%。插入气管导管后，立刻机械通气，在机械通气下判断气管导管位置。采集 B 超数据同前。B 超采用 SonoSite M-Turbo，2-5MHz 凸阵探头。
**疗效评定**	有效性评价指标（主要疗效指标和次要疗效指标）<br>主要指标：气管插管插管后 15min，B 超检查下肺不张的发生率。<br>次要指标：1）术中指标：血压、心率、BIS 等数据的变化；2）苏醒质量的评价；3）术后肺部肺部并发症；4）结局：住院时间，ICU 停留时间，住院费用。
**研究期限**	2019.1.1 – 2022.12.31

一、研究背景

术后肺部并发症（PPCs）严重影响手术病人的术后转归。PPCs 包括：呼吸衰竭（低氧血症）；肺不张；肺部感染；胸腔积液；气胸；支气管痉挛，吸入性肺炎；肺水肿等，其中肺不张占第二位（16.7%），仅次于需要处理的低氧血症的发生率（20.6%）

肺不张指一个或多个肺段或肺叶的容量或含气量减少。全麻诱导期发生率高，约 90%的患者发生肺不张，可持续到术后。全麻诱导后，10%肺组织发生萎陷，重者达到 50%。

肺不张的发生机制主要包括：（1）肺泡陷闭：根据呼吸动力学原理，当肺泡陷闭的力量超过肺泡开放的力量，就会出现肺不张。麻醉后，病人肌肉松弛，膈肌上抬，胸膜腔压力增大，压迫邻近肺组织，不同手术体位，呼吸肌异常，腹内压增高都会增加肺不张的发生率。（2）气体的吸收：众所周知，空气中氮气不会被吸收，可以作为骨架支撑肺泡，不会陷闭。全麻下，吸入氧浓度增加，肺泡氧分压上升，从肺泡进入毛细血管的氧气增加，而作为支架的氮气减少，必然导致肺泡体积减少，而且小气道的闭合也是吸收性肺不张的另外一个原因。闭合后，氧气逐渐被吸收，闭合腔逐渐缩小，直至出现肺不张。（3）表面活性剂异常：肺泡表面活性成分是维持肺泡正常形态的关键，麻醉能抑制表面活性剂的稳定，全麻下，表面活性剂无论是质，还是量都会异常，导致肺泡表面张力的增加和 FRC 的整体减少。

为了确保安全及增加插管期间呼吸暂停的耐受，在全麻前通常有个预充氧过程，成人研究表明 100%吸入氧浓度情况下 3min 预吸氧无论在功能残气量、血液、组织和整个身体都基本达到最佳水平，延长预充氧时间只会增加肺不张的风险。另外，诱导时吸入氧浓度与术后肺不张密切相关，FiO2 60%肺不张发生率只有 0.3%，而 100%的发生率高达 5.6%在预吸氧后 7 分钟肺不张面积开始增加，14 分钟后评价肺不张面积 100%组是 60%组的大约 10 倍，逐渐增加至 45 分钟达到稳定。麻醉中尤其是使用肌松剂后，吸气肌张力消失，相对腹压增大，使膈肌向头部运动，功能残气量下降，肺泡萎陷造成肺不张。

目前为止，尚未有明确的临床研究证明某种单一方法可以有效降低肺不张的发生率。Dongjie Yang 的研究表明单独小潮气量机械通气无效；Meta 分析结果认为 PEEP 对减少肺不张有效；肺复张手法和肺活量手法可以短时间扩张不张肺（7s），但是未有持久研究和术后结果。

成人患者麻醉的研究多集中在潮气量，呼吸频率，吸入氧浓度、PEEP 以及手法或机械膨肺对术后肺不张的影响，现在研究多集中于呼吸参数的研究，但是喉罩与气管插管对肺不张的影响尚无相关研究。

二、研究目的

目前，大量的文献研究表明，术后肺部并发症严重影响手术病人的术后转归，其中肺不张占第二位。肺不张主要发生在全身麻醉诱导建立气道后。在此类手术时，建立不同的气道可能对肺不张的发生有影响，确定哪一种人工气道发生肺不张的比例小，成为医务人员面对此类手术必须要考虑的问题。

本课题在保证患者安全的前提下在并争得患者知情同意的前提下，选择择期进行泌尿，下腹部，下肢手术患者手术的成年患者，进行临床相关数据的采集和观察处理。根据临床及远期随访数据，总结出最佳的预防及治疗术后方案，以供推广。

三、研究设计类型、原则与试验步骤

1. 研究设计

前瞻随机对照研究

患者再采用随机数字法进行随机分组，分为喉罩组和气管插管组。

研究中心：首都医科大学附属北京天坛医院麻醉科。

2. 样本量及研究计划

本研究同为多独立样本均数之间的比较，考虑到边缘误差，将样本量定为每组 90 例。分组情况：氯胺酮组（k 组）和生理盐水组（N 组）。

研究计划：预计氯胺酮组和对照组各 90 例；

2019.2～2019.11 完成一部分临床病例的进行与数据的收集；完成氯胺酮组及对照组，总结部分临床数据；

2019.7～2020.7 完成全部患者的临床病例与数据收集，总结全部临床数据，进行统计分析。撰写论文，参加国际学术交流。

3. 研究期限

2019.2.1- 2020.7.31

4. 受试者选择

入选标准：

1） 年龄 18-60 岁；

2） ASA 1-3 级；

3） 择期全麻下行下腹部泌尿手术、妇科手术、下肢手术患者；

4） 预计手术时间＜2h；

5） 患者及其法定家属已签署知情同意书。

排除标准：

1） 胸部骨折、手术史等应用肺部超声困难者；

2） 术前一月上呼吸道感染史，吸烟史，术前一月全麻或机械通气史；

3） 心功能不全或合并心肺疾病；

4） Mallampati 分级＞II 级。

5） BMI＞30kg/m^2；

6） 有反流误吸可能患者。

四、研究方法

1.分组情况：

患者随机分组，分为喉罩组（L）和气管插管组（E）。

2. 麻醉方法：

所有患者在进入手术室后监测无创血压（BP）、心率（HR）和脉搏血氧饱和度（SPO_2）。术前静脉给予咪达唑仑 2mg，长托宁 1mg。麻醉诱导按主麻医生方案使用：舒芬太尼，异丙酚或依托咪酯，罗库溴铵或不使用。术中机械通气的参数设置为容量控制潮气量 6-8ml/kg，呼吸频率 12-20 次/分钟，吸呼比为 1:1.5，吸入氧浓度 40%。全静脉维持，瑞芬太尼 0.2-0.3 μg/kg/min 泵入，异丙酚 6-8mg/kg/h 泵注，间断给予舒芬太尼和顺阿曲库铵。术中常规监测血压、心率、气道压、呼气末二氧化碳分压（$P_{ET}CO_2$）等。术毕停止异丙酚，瑞芬太尼的输注。术毕统计镇痛药物和麻醉药物的总用量。

3.超声使用：

胸部被分为 12 个象限：前部，侧部和后部区域（由前腋线和后腋线分开），每个区域依据乳头分为右肺和左肺的上部和下部。扫描每个区域的肋间隙，并将每个象限的最病理区域的图像保存为数字格式以进行离线分析。

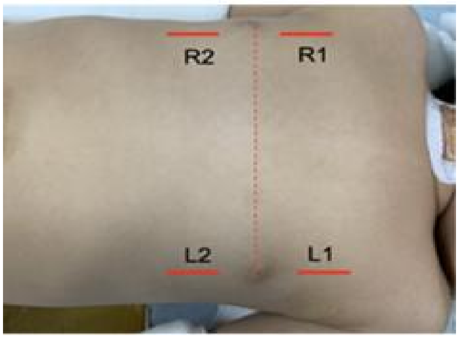

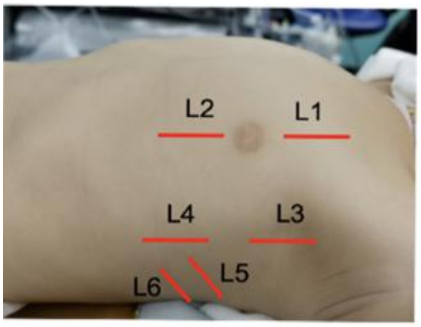

通过计算肺超声评分来评估肺不张。各 12 个象限根据超声评分（指定得分为 0～3 ）。然后通过将 12 个个体象限分数相加来计算 LUS 得分（0-36)，其中得分越高表明通气损失越严重。

原始和改良肺部超声评分：

	正常通气	少量通气损失	中等通气损失	严重通气损失
评分	0	1	2	3
原始肺部超声评分	0-2 B 线	≥3 B 线	多条聚集的 B 线	肺实变
改良肺部超声评分	0-2 B 线	≥3 B 线<br>或<br>由正常胸膜线分隔一个或多个胸膜下实变	多条聚集的 B 线<br>或<br>由增厚或不规则胸膜线分隔的多个胸膜下实变	肺实变 或<br>胸膜下实变直径＞1*2cm

两个肺超声评分可以通过将 12 个单独的肺象限评分相加来计算，得出 0 到 36 之间的评分（没有肺不张）和（完全肺不张）。

4. 干预措施：

喉罩组（L 组）：患者插管前吸 100%氧气。诱导后预充氧时采用机械通气，潮气量 6-8 ml/kg，呼吸频率 12-20 次/分钟，吸入氧浓度 100%。插入喉罩后，立刻机械通气，在机械通气下判断喉罩对位情况。患者入室后，采集肺部 B 超数据。气管插管后 15min 采集肺部 B 超数据。术毕拔除喉罩前采集 B 超数据 。

气管插管组（I 组）患者插管前吸 100%氧气。诱导后预充氧时采用机械通气，潮气量 6-8 ml/kg，呼吸频率 12-20 次/分钟，吸入氧浓度 100%。插入气管导管后，立刻机械通气，在机械通气下判断气管导管位置。采集 B 超数据同前。B 超采用 SonoSite M-Turbo，2-5MHz 凸阵探头。

五、观察指标与检查时间

3 个对结果测量有显著意义的重要时间点如下：T_1，人工气道建立后 15min； T_2，手术结束时；T_3，拔管后 30min。此外，当患者进入手术室(T_0)时，他们将接受基础肺超声评估。

主要结局指标：

气管插管/喉罩置入后 15 min B 超检查下肺不张评分

次要结局指标：

1) T_2、T_3 时 12 个肺区总肺不张评分；
2) T_1、T_2、T_3 时的氧合指数(PaO_2/FiO_2)；
3) 术后 24h 和 48h 气道相关并发症发生率（如声音嘶哑、吞咽困难、喉咙痛等）
4) 术后 24h 和 48h PPCs 发生率术；
5) 术后住院时间。

六、其他需要收集的数据和随访时间

1.患者一般情况的记录： 人口统计学因素：性别、年龄、身高、体重、BMI、ASA、手术种类；

2.术中指标：

①麻醉相关变量：导管型号、建立人工气道呼吸暂停时间、苏醒期有无躁动、循环情况、麻醉药量、麻醉时间、停药后到拔除人工气道时间、出入量。

②术中各系统指标：

①麻醉及气道相关变量：喉罩/气管导管型号、建立人工气道呼吸暂停时间、苏醒期有无躁动、循环情况、麻醉药量、麻醉时间、停药后到拔除人工气道时间、出入量。

②术中各系统指标：

A．呼吸系统：SPO_2、$ETCO_2$、机械通气参数（潮气量，呼吸频率，PEEP），峰值吸气压力，吸气末平台压力。

B．循环系统：血压、心率。

3 .随访时间：

术后 24h、48h 气道并发症：喉咙痛、吞咽困难、发音异常。

术后 24h、48h 肺部并发症：肺炎、体温、白细胞、是否需要治疗。

七、项目风险的预评估及风险处置预案

因为本研究涉及到的主要处理措施就是术中选择气管插管或喉罩，其他均为临床常规处置，这两种人工气道都是此类手术常用的人工气道，因此本研究一般来说本研究不存在风险。术中及术后应用 B 超检查对患者是无害的。

八、资料保存和保密

病例报告表，资料的记录和保存。知情同意书将包括一个声明，即患者允许已授权的申办方、伦理委员会、权威机构直接查阅病例报告表上相关的原始资料（如患者的医疗档案、预约记录、 原始实验室记录等)。研究人员应遵循职业保密规定，必须对患者的所有个人身份信息或医疗信息保密。

九、数据安全监查

临床研究将根据风险大小制定相应的数据安全监察计划。所有不良事件均详细记录，

恰当处理并追踪直到妥善解决或病情稳定，按照规定及时向伦理委员会、主管部门、申办者和药品监督管理部门报告严重不良事件与非预期事件等；主要研究者定期对所有不良事件进行累积性回顾，必要时召开研究者会议评估研究的风险与受益；双盲试验必要时可以进行紧急揭盲，以确保受试者安全与权益；大于最小风险的研究将安排独立的数据监查员对研究数据进行监查，高风险研究将建立独立的数据安全监察委员会对累积的安全性数据以及有效性数据进行监查，以做出研究是否继续进行的建议。

十、统计学处理

本研究将使用 SPSS 软件进行统计分析。连续变量将以平均±标准差(x±s)介质或四分位数范围表示，分类变量将以数字（比例，%）表示。将进行柯尔莫戈罗夫-斯米尔诺夫检验来检测连续变量的正态分布。在进行连续变量的正态性检验后，将使用 t 检验、方差分析或曼-惠特尼检验进行组间和组内的适当比较。卡方检验和 Fisher 精确检验将用于分析分类变量。所有的测试都将是双尾的，并在 5%的显著性水平上进行。统计学意义为 P<为 0.05。丢失主要结果的参与者将被排除在结果分析之外，如果意外退出率超过 10%，将进行多重插补。

十一、伦理声明

在研究开始之前，由伦理委员会批准该试验方案后才实施临床研究。这次研究严格遵守最新的《赫尔辛基宣言》的原则

十二、参加人员

姓名	职称/专业	任务
金旭	主任医师/麻醉学	研究设计，研究管理
李学斌	主治医师/麻醉学	研究设计，临床麻醉
刘彬	主治医师/麻醉学	超声评估
王雅欣	硕士研究生/麻醉学	数据收集与管理
熊蔚	主治医师/麻醉学	病人招募
张园	主治医师/麻醉学	临床麻醉

鲍迪	硕士研究生/麻醉学	患者随访
梁艺	硕士研究生/麻醉学	临床注册，伦理申请

首都医科大学附属北京天坛医院医学伦理委员会

IRB of Beijing Tiantan Hospital, Capital Medical University

伦理审查意见

IRB Review Suggestions

Ethical approval number：KY 2019-006-01

伦理审查编号	KY 2019-006-01
项目全称（编号） Full name of the project:	喉罩对比气管插管用于全麻手术肺不张的随机对照研究 Laryngeal mask airway versus endotracheal tube for atelectasis: a protocol for a randomized controlled trial
申办者/CRO Main research institutes:	首都医科大学附属北京天坛医院 Beijing Tiantan Hospital, Capital Medical University
主要研究者/所在科室 Principal investigator /main research department:	金旭/麻醉科 Xu Jin / Department of Anesthesiology
审查日期 Review date:	2019-01-09 January 09, 2019
审查地点 Review site:	首都医科大学附属北京天坛医院 Beijing Tiantan Hospital, Capital Medical University
审查文件	临床试验初次伦理审查申请 Application for initial ethical review<br>伦理申请专家科研论证表 Scientific research demonstration form<br>喉罩 科研项目临床研究方案(V3.0/2019-01-01) Clinical research protocol V3.0<br>插管-喉罩 CRF（V3.0） Case report form V3.0<br>插管-喉罩标书 Biding document<br>知情同意书(V1.0/2018-10-1) Informed consent V1.0<br>研究者简历及 GCP 培训证书 Resume of principal investigator, list of participants, and GCP certificate
伦理审查方式 Ethical review type:	■快速审查 Rapid review □会议审查
审查委员	王凯戎、罗芳

Review comments:

审查意见：

根据我国卫生部《涉及人的生物医学研究伦理审查办法（试行）》（2007）、《医疗技术临床应用管理办法》（2009），国家食品药品监督管理局（CFDA）《药物临床试验伦理审查工作指导原则》（2010）、《药物临床试验质量管理规范》（2003）、《医疗器械临床试验质量管理规范》（2016），世界医学会《赫尔辛基宣言》，以及国际医学科学组织委员会《人体生物医学研究国际道德指南》的伦理原则，经本伦理委员会审查，意见如下：

同意按照研究方案进行关于喉罩对比气管插管用于全麻手术肺不张的随机对照研究

复审审查方式	■快速审查 □会议审查

请对研究方案文件/知情同意书等文件根据上述意见做出修改/补充，并将修改/补充文件在6个月内提交伦理委员会审查。如果对审查意见有不同观点，请书面向伦理委员会主任委员反映。

主任委员签字	[signature]	日期	2019-01-09

首都医科大学附属北京天坛医院医学伦理委员会（盖章）

扫描全能王 创建
