# Supplementary material for: The effects of laryngeal mask airway versus endotracheal tube on atelectasis in patients undergoing general anesthesia assessed by lung ultrasound: A protocol for a prospective, randomized controlled trial
Source: PLoS One. 2022 Sep 9;17(9):e0273410. doi: 10.1371/journal.pone.0273410 (PMC9462747; doi:10.1371/journal.pone.0273410)

Research scheme

The Effects of Laryngeal Mask Airway Versus Endotracheal Tube on Atelectasis in Patients Undergoing General Anesthesia:
A protocol for a prospective, randomized controlled trial

CRO： Beijing Tiantan Hospital,Capital Medical University

Version: 3.0

Version date: 2020.01.01

Study duration: 2019.2-2022.12

Principal investigator: Xu Jin

Department: Anesthesiology department

Research protocol summary

**Project name**	The Effects of Laryngeal Mask Airway Versus Endotracheal Tube on Atelectasis in Patients Undergoing General Anesthesia: A protocol for a prospective, randomized controlled trial
**Research objective**	The incidence of atelectasis is high in patients undergoing general anesthesia. This may further damage oxygenation and cause postoperative pulmonary complications (PPCs). The advantages of lung-protective ventilation strategy in reducing atelectasis have been confirmed; however, with the increasing application of laryngeal mask airway (LMA), there is hardly any relevant study to compare the effects of LMA and endotracheal tube (ETT) on atelectasis to date. Additionally, lung ultrasound has been increasingly used for bedside atelectasis diagnosis. For the above considerations, we designed this trial to compare the effects of LMA and ETT on atelectasis assessed using lung ultrasound scores (LUS). This study provides more powerful clinical evidence for perioperative respiratory management.
**Design**	Prospective, Randomized, Controlled Double-blind study
**Sample size**	180 patients
**Eligibility criteria**	**Inclusion criteria**<br>1.Patients ≥18 years old and < 60 years old;<br>2.(ASA) physical status of I–III;<br>3.Patients scheduled for elective lower abdominal surgery;<br>4.Estimated operation time is < 2 h;<br>5.Patients and/or their authorized surrogates have signed the informed consent.
	**Exclusion criteria**<br>1.Patients have difficulty with lung ultrasound, such as chest fractures, thoracic deformity, and thoracic surgical history;<br>2.Patients with a history of upper respiratory tract infection, smoking, general anesthesia, or mechanical ventilation in the first month before surgery;<br>3.Patients with cardiac insufficiency (New York Heart Association class IV), chronic renal failure (glomerular filtration rate < 30 mL $min^{-1} \cdot 1.73\ m^{-2}$), liver cirrhosis (Child B or C), or pregnancy;<br>4.Patients with airway abnormalities or reactive airway diseases, including Mallampati classification > II;

	5.Patients with BMI>30 kg/m^2;<br>6.Patients with the risk of reflux aspiration.
**Intervention**	Group L: In this group, LMA are selected for the artificial airway to maintain mechanical ventilation during general anesthesia. LMA size depends on the weight of the patients. After anesthesia induction and 3 min pre-oxygenation, the LMA will be inserted at the appropriate position and fixed properly.<br>Group E: In this group, ETT are selected for the artificial airway to maintain mechanical ventilation during general anesthesia. Normally, men and women choose a tube with the inner diameter (ID) = 8 mm, and 7.5 mm, respectively. Similar to Group L, ETT will be intubated with appropriate depth and fixed properly in the trachea after anesthesia induction.
**Outcome measurement**	Three time points were significant for outcome measurement, including 15 min after the establishment of the artificial airway (T_1), the end of surgery (T_2), and 30 min after extubation (T_3). In addition, when patients enter the operation room (T_0), they will accept the lung ultrasound assessment for the basic LUS.<br><br>**Primary outcome:** Total atelectasis LUS of 12 lung regions at T_1.<br><br>**Secondary outcomes:**<br>1) Total atelectasis LUS of 12 lung regions at T_2, T_3;<br>2) Oxygenation index (PaO_2/FiO_2) at T_1, T_2, T_3;<br>3) Incidence of postoperative airway complications such as hoarseness, dysphagia, and sore throat during 24 h and 48 h after surgery<br>4) The incidence of PPCs during 24 h and 48 h after surgery;<br>5) Length of stay.
**Study duration**	2019.2-2022.12

1. Background

For patients undergoing general anesthesia, atelectasis can occur a few minutes after pre-oxygenation and last until after surgery, further contributing to the incidence of other postoperative pulmonary complications (PPCs), increasing mortality, morbidity, and length of stay. The mechanism of atelectasis during general anesthesia has not been fully clarified, which may be related to absorption atelectasis. Airway secretion can be trapped in patients undergoing mechanical ventilation, which may block distal airway obstruction. In this situation, particularly for high FiO_2, gas inflow is blocked and gas uptake by the blood continues, which can lead to alveolar collapse and promote the formation of atelectasis.

Previous studies have demonstrated that lung-protective ventilation strategies can effectively improve atelectasis and reduce the incidence of PPCs. However, there is hardly any relevant study to compare the effects of different artificial airways on atelectasis. The endotracheal tube (ETT) has always been considered the gold standard for ensuring a patent airway and adequate ventilation during general anesthesia. The supraglottic airway device, laryngeal mask airway (LMA), is increasingly used for ventilation maintenance during general anesthesia as it is more comfortable with less irritation and lower airway resistance. In recent years, the application of LMA in surgery has become increasingly common. However, LMA cannot completely isolate the airway, and inadequate ventilation due to gas leakage could result in a low ventilation-perfusion ratio, which may lead to atelectasis. Therefore, the effects of LMA vs. ETT on atelectasis in patients undergoing general anesthesia require further investigation.

2. Objective

This prospective, single-center, single-blinded, randomized controlled trial aimed to compare the effects of LMA and ETT on atelectasis in patients undergoing general anesthesia assessed by lung ultrasound and to compare the prognosis of LMA and ETT.

3. Study design

(1) Study type

This study is a prospective, single-center, single-blinded, randomized controlled trial. The 180 patients who meet the inclusion and exclusion criteria will be randomly allocated to the ETT and LMA groups. This trial is designed according to the updated Consolidated Standards of Reporting Trials statement and conducted at Beijing Tiantan Hospital, Capital Medical University from February 2019 to December 2022.

(2) Sample size calculation

Our pre-tests estimated that the atelectasis LUS in ETT was 8.2±3.87. The difference in LUS, at least for a score of 2, is considered clinically significant. The alpha level was set at 0.05, the beta value was set to 0.1, and allowing for a dropout

rate of 10%. We calculated that the total sample size required 180 patients (90 patients in each group).

(3) Study duration

2019.2-2022.12

(4) Eligibility

Inclusion criteria

1) Patients ≥18 years old and < 60 years old;
2) American Society of Anesthesiologists (ASA) physical status of I–III;
3) Patients scheduled for elective lower abdominal surgery;
4) Estimated operation time is < 2 h;
5) Patients and/or their authorized surrogates have signed the informed consent.

Exclusion criteria

1) Patients have difficulty with lung ultrasound, such as chest fractures, thoracic deformity, and thoracic surgical history;
2) Patients with a history of upper respiratory tract infection, smoking, general anesthesia, or mechanical ventilation in the first month before surgery;
3) Patients with cardiac insufficiency (New York Heart Association class IV), chronic renal failure (glomerular filtration rate <30 mL min−1·1.73 m−2), liver cirrhosis (Child B or C), or pregnancy;
4) Patients with airway abnormalities or reactive airway diseases, including Mallampati classification > II;
5) Patients with BMI>30 kg/m2;
6) Patients with the risk of reflux aspiration.

4. Method

(1) Allocation

A hundred and eighty eligible patients will be randomly assigned to the LMA group (Group L) and ETT group (Group E) at a 1:1 ratio.

(2) Anesthesia management

Routine monitoring will be performed after participants enter the operating room, including electrocardiogram, non-invasive blood pressure (BP), pulse oxygen saturation (SpO_2), bispectral index, and temperature. Intravenously administered 0.025–0.075 $mg\cdot kg^{-1}$ midazolam as a premedication. 0.3–0.5 $\mu g\cdot kg^{-1}$ sufentanil, 1.5–2.5 $mg\cdot kg^{-1}$ propofol, and 0.15 $mg\cdot kg^{-1}$ cisatracurium will be administrated for anesthesia induction. After 3 min of pre-oxygenation, the anesthesiologist in charge will establish an artificial airway according to the allocation information and cover an asepsis scarf to ensure that the intervention is blinded to the lung ultrasound assessor. Anesthesia maintenance will be administered with a total intravenous infusion of 6–8 $mg\cdot kg^{-1}\cdot h^{-1}$ propofol and 0.2–0.3 $\mu g\cdot kg^{-1}\cdot min^{-1}$ remifentanil according to patients' circulation conditions and anesthesia depth. Mean arterial pressure (MAP) and heart

rate (HR) will remain within 30% of the baseline level, and the temperature will remain within 36–37°C during operation. If necessary, vasoactive agents will be used to maintain stable circulation, and the agent type, dosage, and use time will be recorded accurately. Lung protective ventilation strategy will be applied during operation and related parameters are set as follows: tidal volume (Vt) at 6–8 $mL\cdot kg^{-1}$, PEEP=5 cmH_2O, with recruitment maneuver (in total of three times, each time with a pressure of 30 cmH_2O for 10 s), I:E=1:1.5, FiO_2 at 0.4, oxygen flow rate at 1.5 L/min, and respiratory rate (RR) at 12–20 bpm to maintain the end-tidal carbon dioxide partial pressure ($P_{ET}CO_2$) range from 35 mmHg to 45 mmHg. All anesthetic infusions will be discontinued at the end of surgery. The postoperative transfer location, including the postoperative anesthesia care unit or intensive care unit, will be decided together by the anesthesiologist and surgeon. Flurbiprofen axetil intravenously or acetaminophen orally are administered for necessary postoperative analgesia, and the dose and time of administration will be accurately recorded.

(3) Lung ultrasound

Lung ultrasound assessment will be performed by a blinded trained anesthesiologist. As shown in a previous study, ultrasound scanning will select a 5–12 MHz linear transducer (SonoSite M-Turbo) to parallelly scan all the intercostal space of 12 regions from right to left, anterior to posterior of patients' chest in the supine position. The six regions are divided by anterior midline, clavicular midline, anterior axillary line, posterior axillary line, and pectoral nipple line in each hemithorax. This anesthesiologist will complete the assessment according to the LUS criteria at the same time as scanning, and the significant images and videos will be recorded.

Atelectasis LUS criteria: 0 indicates normally ventilated areas with lung sliding sign: A-line, or isolated B-lines (< 3); 1 indicates a mild reduction in lung ventilation/moderate loss of lung tissue gasification; B lines (≥ 3) with clear boundary, regular distribution, and spacing ≥ 7 mm, or irregular, clear interval; 2 indicates a severe reduction in lung ventilation/severe lung tissue gasification: multiple coalescent B-lines spaced ≤ 3 mm and continuous fusion; 3 indicates atelectasis/pulmonary consolidation: tissue-like signs, fragment signs, and bronchial inflation signs.

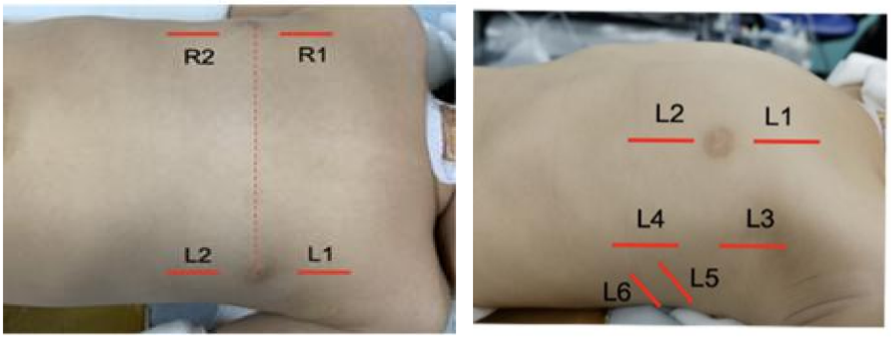

(4) Intervention

Group L: In this group, LMA are selected for the artificial airway to maintain mechanical ventilation during general anesthesia. LMA size depends on the weight of the patients. After anesthesia induction and 3 min pre-oxygenation, the LMA will be inserted at the appropriate position and fixed properly.

Group E: In this group, ETT are selected for the artificial airway to maintain mechanical ventilation during general anesthesia. Normally, men and women choose a tube with the inner diameter (ID) = 8 mm, and 7.5 mm, respectively. Similar to Group L, ETT will be intubated with appropriate depth and fixed properly in the trachea after anesthesia induction.

5. Outcomes

Three time points were significant for outcome measurement, including 15 min after the establishment of the artificial airway (T_1), the end of surgery (T_2), and 30 min after extubation (T_3). In addition, when patients enter the operation room (T_0), they will accept the lung ultrasound assessment for the basic LUS.

Primary outcome: Total atelectasis LUS of 12 lung regions at T_1.

Secondary outcomes:

1) Total atelectasis LUS of 12 lung regions at T_2, T_3;
2) Oxygenation index (PaO_2/FiO_2) at T_1, T_2, T_3;
3) Incidence of postoperative airway complications such as hoarseness, dysphagia, and sore throat during 24 h and 48 h after surgery
4) The incidence of PPCs[26] during 24 h and 48 h after surgery;
5) Length of stay.

6. Data collection and follow-up

The indicators that were recorded included baseline data, perioperative data, and follow-up data. All records can be found in the case report form (CRF).

Baseline data included: 1) Participants' general information such as age, height, weight, and vital signs such as basic HR, MAP, RR, and SpO_2; 2) ASA physical status; 3) Mallampati status; 4) preoperative complications; and 5) preoperative laboratory parameters.

Perioperative data included: 1) Vital signs (HR, MAP, RR, and SpO_2) will be recorded every 15 min from the beginning of the operation to 30 min after extubation; 2) Mechanical ventilation parameters (Vt, plateau pressure [P_{plat}], airway peak pressure [P_{peak}], and dynamic lung compliance [Cdyn] [Cdyn = tidal volume/{PIP-PEEP}]) will be recorded every 15 min from the beginning of the operation to extubation; 3) Atelectasis LUS at T_0, T_1, T_2, and T_3; 4) Arterial blood-gas analysis results at T_0, T_1, T_2, and T_3; 4) Administration of perioperative medicines, complete with the type, dosage, way, and time; 5) Several important durations, including the duration of anesthesia, operation, mechanical ventilation duration, and

extubation; and 6) Intraoperative quantity of liquid intake, urine, and blood loss.

Follow-up will be performed by the blinded and trained researchers at 24 h and 48 h. Postoperative complications, length of stay, and adverse events during hospitalization will be recorded in detail in the CRF.

7. Harm and solution

If adverse events or serious adverse events (such as reflux aspiration, serious leakage of LMA, severe circulatory instability) occur intraoperatively or postoper

atively, PI and the Institutional Review Board (IRB) will be informed immediately. The IRB and PI have the right to break the blinding, if necessary.

8. Data preservation and confidentiality

Participants will be asked whether relevant data can be used when signing informed consent. Each patient will be assigned a unique study identifier after they are recruited in the study to conceal and protect private information. This trial does not involve the collection of biological specimens for storage. All paper materials will be stored in the locked cabinet of the anesthesiology department of Beijing Tiantan Hospital. The electronic data will be stored in the database. Only PI has access to all information. If the researchers in charge of the result analysis need access, they need to apply access permission from PI, and access time will be recorded in detail.

9. Data motoring

The data management committee consists of PI, and two statisticians will provide monitoring, including verifying the progress of the study and all observational data. Quality control was performed at all stages of the data processing. There is no interim analysis plan for our study because of the low-risk intervention and short-term research periods.

10. statistical analysis

Statistical analysis will be performed using SPSS software (version 23.0; International Business Machines Inc., USA). Continuous variables will be presented as mean ± standard deviation (x±s) media, or interquartile range, and categorical variables will be presented as numbers (proportion, %). Kolmogorov–Smirnov tests will be performed to detect the normal distribution of continuous variables. After testing for normality of continuous variables, the Student's t-test, analysis of variance, or the Mann-Whitney U-test will be used for appropriate comparisons between and within groups. Chi-squared and Fisher's exact tests will be used to analyze categorical variables. All tests will be two-tailed and conducted at a 5% significance level. Statistical significance will be set at $P<0.05$. The participants missing the primary outcome will be excluded from the outcome analysis, and multiple imputations will be conducted if the unintended drop-out rates are more than 10%.

11. Ethical statement

The trial strictly adhere to the principles of the latest Declaration of Helsinki.

12. Participants

Name	Positional title	Assignment
Xu Jin	Chief physician	Study design and management; Clinical anesthesia
Xuebin Li	Attending doctor	Study design; Lung ultrasound assessment
Bin Liu	Attending doctor	Lung ultrasound assessment; Manuscript writing;
Yaxin Wang	Postgraduate student	Manuscript writing; Data collection and management
Wei Xiong	Deputy chief physician	Patients recruitment
Yuan Zhang	Attending doctor	Clinical anesthesia
Di Bao	Postgraduate student	Follow-up
Yi Liang	Postgraduate student	Clinical registration; Ethical application

IRB of Beijing Tiantan Hospital, Capital Medical University

IRB Review Suggestions

**Ethical approval number**	KY 2019-006-01
**Full name of the project**	Laryngeal mask airway versus endotracheal tube for atelectasis: a protocol for a randomized controlled trial
**Main research institutes**	Beijing Tiantan Hospital, Capital Medical University
**Principal investigator /main research department**	Xu Jin / Department of Anesthesiology
**Review date**	January 09, 2019
**Review site**	Beijing Tiantan Hospital, Capital Medical University
**Review documents**	Application for initial ethical review<br>Scientific research demonstration form<br>Clinical research protocol V3.0<br>Case report form V3.0<br>Biding document<br>Informed consent V1.0<br>Resume of principal investigator, list of participants, and GCP certificate
**Ethical review type**	Rapid review
**Reviewer**	Kairong Wang, Fang Luo

Review comments:

The prospective, randomized, double-blinded, controlled study for *Laryngeal mask airway versus endotracheal tube for atelectasis* was approved according to the clinical research scheme

If you have different opinions on the review opinions, please write to the IRB

**Signature of chairman**	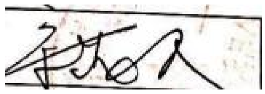	**Date**	**2019-01-09**

IRB of Beijing Tiantan Hospital, Capital Medical University (seal)

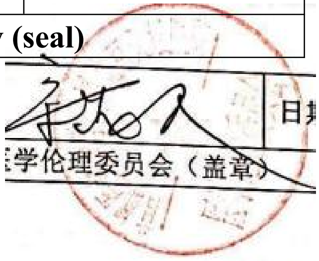

Supplement: S3 File — (PDF) [file pone.0273410.s003.pdf]
